# A Rare Case of Spasm of the Voice

**Published:** 1883-01

**Authors:** 


A Rare Case of Spasm of the Voice.—Spasm of the glottis in its more unusual aspects recently received quite a number of additional cases. None of them, however, equal in interest that recorded by Strassmann occurring in the Medical Clinic of Jena.

A young girl, aged 16, who had not yet menstruated, of healthy parentage, and who had been, up to this time, in good health herself, noticed on the 21st of December that with every breath there was a voice—a sound—which she could not control or stop.

This "croak"—for it resembled the croak of a frog—varied in intensity at different periods of the day; it continued during her sleep, but otherwise was not influenced by exercise or any endeavors on her part to vary it. There was no pain, no dyspnœa, and "her own voice" could be used at will and independent of the other. She was taken to Prof. Nothnagel. Her condition then was as follows: a slim, rather undeveloped girl, healthy looking, but slightly strumous. The voice within could be heard by those around her; it was not increased by deep inspiration, nor by weeping or talking. The cervical muscles normal in action; the laryngeal box with every one of the "sounds" was drawn downwards; respiration, 20; pulse-rate, 64 and regular.

Bromide of potassa, the galvanic current, and the horse-shoe magnet were remedies that proved of no avail.

Then metallotherapy was tried. A piece of silver was placed, without any results, on either side of the larynx. A little plate of copper was then applied. This effected the cure.

There was a neurosis whose seat was in the inferior laryngeal nerve. That other pneumogastric branches were involved was evidenced by irregular, and at times, rapid heart action. There was spasm—cramp—of the adductors of the glottis, and this was, besides, an inspiratory spasm. Strassmann says further that it is a "matter of taste" whether an hysterical origin be ascribed or not.—*Ber. Klin. Woch.*, No. 46, 1882.

